# Supplementary figures and images for: Genome-wide identification and expression reveal the involvement of the FCS-like zinc finger (FLZ) gene family in Gossypium hirsutum at low temperature
Source: PeerJ. 2023 Jan 23;11:e14690. doi: 10.7717/peerj.14690 (PMC9879155; doi:10.7717/peerj.14690)

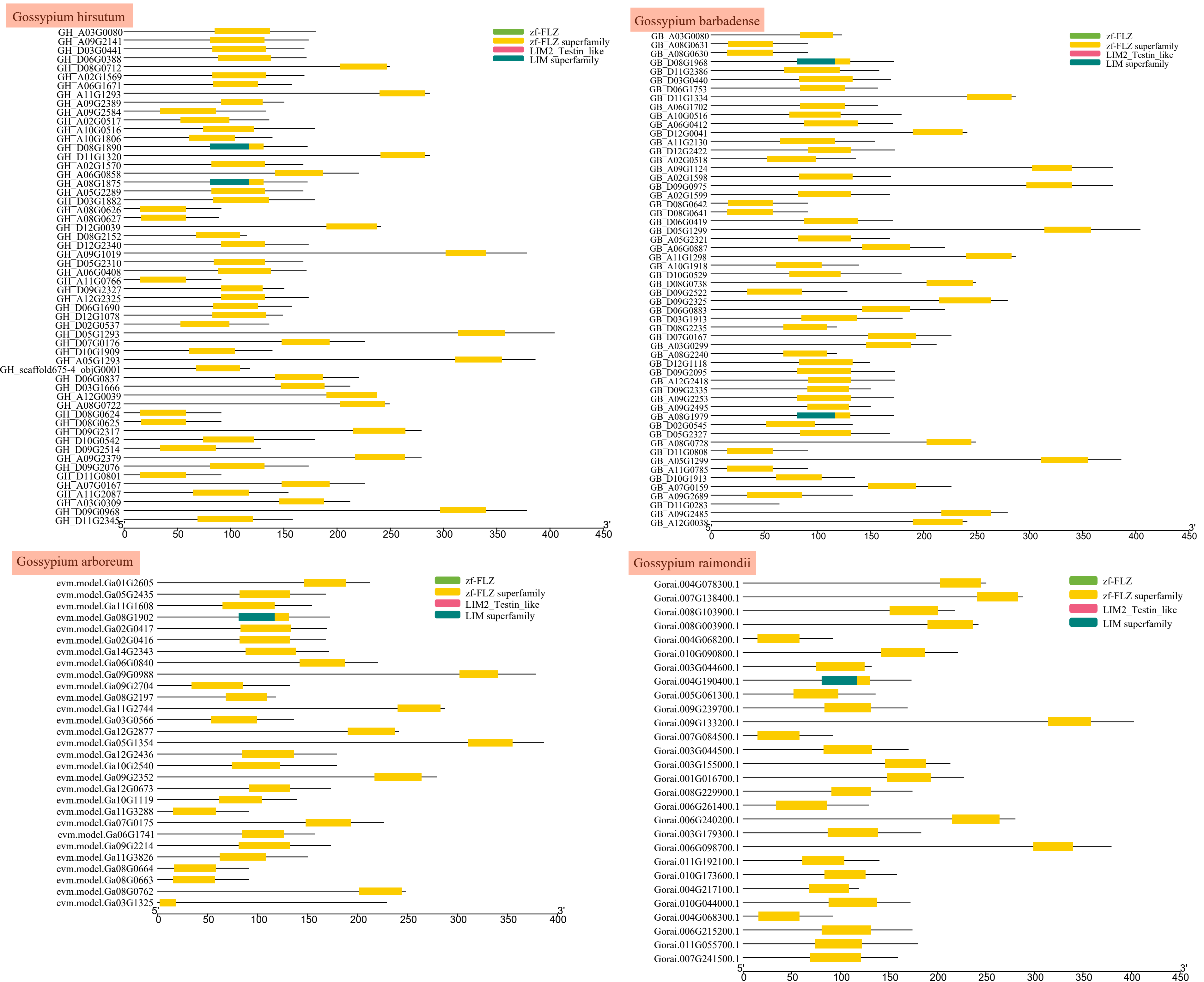

Fig S1.Distribution of the FLZ domain in the FLZ proteins of cotton.

Supplement: Supplemental Information 1 [file peerj-11-14690-s001.pdf]

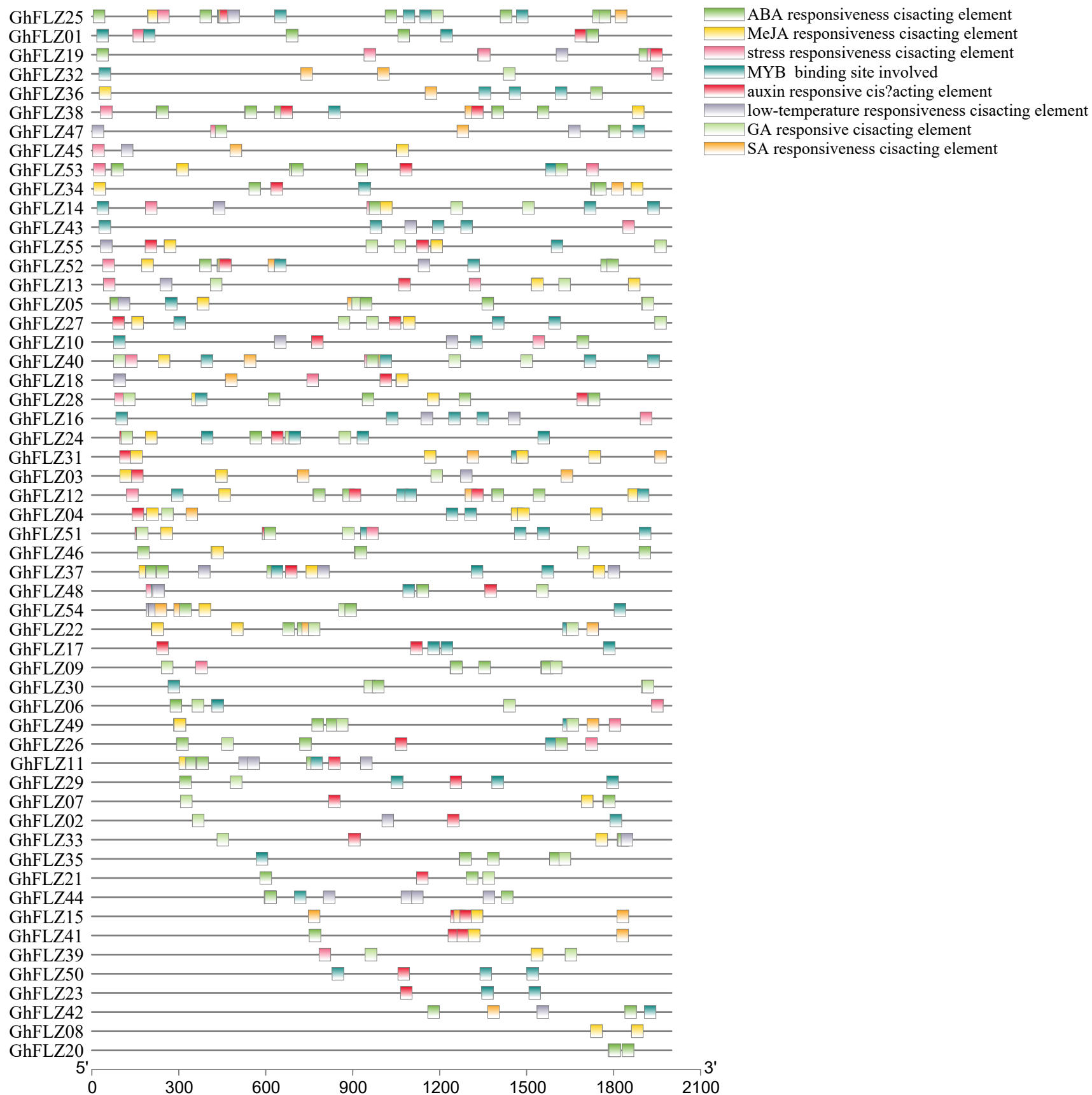

Supplement: Supplemental Information 2 [file peerj-11-14690-s002.pdf]
