# Supplementary material for: Genome-wide identification and expression reveal the involvement of the FCS-like zinc finger (FLZ) gene family in Gossypium hirsutum at low temperature
Source: PeerJ. 2023 Jan 23;11:e14690. doi: 10.7717/peerj.14690 (PMC9879155; doi:10.7717/peerj.14690)
Supplement: Supplemental Information 3 [file peerj-11-14690-s003.docx]

**Table S1. All primers used in this study**

| gene_name | Forward primer | Reverse primer |
| --- | --- | --- |
| GhFLZ03 | GCCGATCAAGAGGACGACAA | AGCGTTGATCGTAACGGGAG |
| GhFLZ07 | TCACGTCAGCCGATCAAGAG | GGGGGATTCATGCAAAGGGT |
| GhFLZ11 | AGGTCGACCCATGATCGGAA | GTGGACTTTTGGGGCTCTT |
| GhFLZ24 | AGGTCTTGTTTGGGACCGAG | GGCATCCATTGAAGACCCGA |
| GhFLZ25 | GTTGTCGCCAACAAGTGCAA | CCAAGCCGCGATTACAAAGG |
| GhFLZ33 | CGAGGCAACCGATGAAGAGA | GCCAAGGACCGCTGATCTAA |
| GhFLZ37 | AGGTCGACCCATGATCGGAA | GTGGACTTTTGGGGCTCTT |
| GhFLZ44 | ATGTACAGAGGGACAACCT | CAACAGTGCCTGGTCGGAAA |
| GhFLZ45 | AGGTGCAAAAGAAGCCCACT | GGCTTCAGGTTCTGGGGTTT |
| GhFLZ50 | TCTTTGTCGCAAACCGCTTC | CCGGTCCGTACAGCTTTGTT |
| GhFLZ51 | CGATGCCTGCTCTCTTTGTC | TAGGAGTGGAGTTTCTGGGCAT |
| GhFLZ55 | GGAGTCTGGGTATGCAGGAAT | ACCTTGGAGAAGAAGCCGAA |
